# Can Global Weed Assemblages Be Used to Predict Future Weeds?

**DOI:** 10.1371/journal.pone.0055547

**Published:** 2013-02-05

**Authors:** Louise Morin, Dean R. Paini, Roderick P. Randall

**Affiliations:** 1 Commonwealth Scientific and Industrial Research Organisation (CSIRO), Ecosystem Sciences, Biosecurity Flagship, Canberra, Australian Capital Territory, Australia; 2 Department of Agriculture and Food Western Australia, Perth, Western Australia, Australia; University of Tartu, Estonia

## Abstract

Predicting which plant taxa are more likely to become weeds in a region presents significant challenges to both researchers and government agencies. Often it is done in a qualitative or semi-quantitative way. In this study, we explored the potential of using the quantitative self-organising map (SOM) approach to analyse global weed assemblages and estimate likelihoods of plant taxa becoming weeds before and after they have been moved to a new region. The SOM approach examines plant taxa associations by analysing where a taxon is recorded as a weed and what other taxa are recorded as weeds in those regions. The dataset analysed was extracted from a pre-existing, extensive worldwide database of plant taxa recorded as weeds or other related status and, following reformatting, included 187 regions and 6690 plant taxa. To assess the value of the SOM approach we selected Australia as a case study. We found that the key and most important limitation in using such analytical approach lies with the dataset used. The classification of a taxon as a weed in the literature is not often based on actual data that document the economic, environmental and/or social impact of the taxon, but mostly based on human perceptions that the taxon is troublesome or simply not wanted in a particular situation. The adoption of consistent and objective criteria that incorporate a standardized approach for impact assessment of plant taxa will be necessary to develop a new global database suitable to make predictions regarding weediness using methods like SOM. It may however, be more realistic to opt for a classification system that focuses on the invasive characteristics of plant taxa without any inference to impacts, which to be defined would require some level of research to avoid bias from human perceptions and value systems.

## Introduction

The movement of vascular plants across regions has always occurred, primarily with human assistance, and has increased exponentially in recent times with increases in global trade [1], [2]. Some plants have been accidentally introduced to new regions as contaminants of various merchandises and seed lots. Most plants however, have been deliberately moved because of their perceived desirable attributes as a source of food, fodder or fibre, or for aesthetic (ornamental) reasons. The majority of plant introductions have either been beneficial or of no particular value, but a fraction of these plants have caused problems in their new range or have the potential to do so in the future (e.g. [3]).

Plants, native or introduced, that grow in unwanted sites and often have detectable detrimental economic and/or environmental consequences are commonly referred to as weeds [4], [5]. In an attempt to address confusion with the plant naturalization/invasion process, Richardson et al. [4] made a solid attempt to better define the terminology employed. According to these authors the terms weed and invasive plant are not interchangeable, because the latter does not infer any environmental and economic impact. This proposed terminology however, has not been widely applied and confusion continues to abound in the literature. Weeds can be a major constraint to agricultural production and a key threat to the integrity and sustainability of natural ecosystems [6], [7]. Large sums of money are invested worldwide to manage them (e.g. [8], [9], [10]) and there is increasing interest in identifying potential major weeds of the future and managing them early [11], [12].

Determining which plant taxon is more likely to become a weed in a particular region is a challenge because it depends on attributes of the taxon and the invasibility of communities, habitats and regions [13], [14]. While plant invasiveness predictions have been reliable within some plant families [15], there appears to be no specific plant trait that can be collectively applied to all taxa to predict invasiveness and even less to predict ‘weediness’ (aka Richardson et al. [4] definitions) [16], [17]. Three broad risk assessment approaches – quantitative statistical models, semi-quantitative scoring and qualitative expert assessment – have been developed to screen plant taxa for potential weediness [18], [19], [20]. This type of screening has primarily been used as part of quarantine protocols to decide whether or not importation of a new plant taxon should be allowed in a country. The Australian Weed Risk Assessment approach is a semi-quantitative scoring system based on a series of questions on a plant's biology, biogeography, undesirable attributes and behaviour elsewhere [21]. It has been the most widely considered and implemented risk assessment methodology [22], [23], [24]. This approach however, is not without its critics because of its high level of uncertainty and inaccuracy, as well as the potential cognitive biases of assessors [20], [25], [26], and recommendations have been put forward to improve the process [27], [28], [29], [30]. Expert opinions and structured weed risk assessment have also been used to identify which of the already introduced plant taxa in a region (including ‘sleeper weeds’ *sensu* Groves [12]) are more likely to become weeds in the future to assist with management prioritization (e.g. eradication, containment) [11], [31], [32]. While experts will have an in-depth knowledge of a potentially large range of plant taxa, this knowledge is unlikely to encompass all the introduced taxa. In addition, expert solicitation has been found to be prone to a range of cognitive biases such as past experience, overconfidence, motivational bias, and lack of independence [33].

Considering the many thousands of plant taxa that exist around the globe, the ability to perform a simultaneous analysis of a large number of taxa to predict weediness in particular regions would be most valuable. This is an attractive proposition because it could provide an initial screening of potential weeds and bring down numbers to a more manageable level for subsequent in-depth risk analyses. Self-organising maps (SOMs) have been used to analyse assemblages of non-native fish species, insect pests and fungal plant pathogens to predict likelihood of establishment of species in particular regions using presence/absence data [34], [35], [36], [37], [38]. They have been shown to be robust at distinguishing between those species that can establish and those that cannot [38] and resilient to significant errors in the dataset [37]. Paini et al. [38] tested the SOM approach in a virtual world of invasive species and revealed that it was able to consistently rank a high percentage of those species that could establish above those that could not. On the other hand, alterations of up to 20% of global insect crop pest data in a dataset analysed with a SOM demonstrated that it did not make a difference in the ranking of species according to their ‘risk’ of establishing in a particular region [37].

A similar SOM approach could be used to analyse assemblages of weeds to predict the likelihood of plant taxa becoming weeds in a new region [30]. The central tenet of this approach is that the weed assemblage of a region captures a significant proportion of the biological, ecological and abiotic factors of that region, which may not be easily measured. The SOM would cluster regions based on the weed assemblage and use this clustering information to rank plant taxa by their likelihood to become weeds. Put simply, if one region has weed taxa 1–10, and a second region has weed taxa 1–9, then it would be reasonable to assume that weed taxon 10 has a high likelihood of becoming a weed in the second region, given its association with weed taxa 1–9 in the first region. The SOM is capable of making this type of comparison except with all taxa and all regions simultaneously.

In this study we used a dataset extracted from a pre-existing, extensive worldwide database of plant taxa recorded as weeds or other related status [39], [40] and performed a SOM analysis to explore the potential of this species association approach in predicting weediness of plant taxa across the globe. This database is currently the best available and most extensive compilation of information on the status of plants cited in more than 1,000 sources from different regions of the world. It is noteworthy that the various plant status categories in the database originate from the sources consulted and since their veracity cannot be vouched for they cannot be aligned with those proposed by Richardson et al. [4]. The same database has been previously used in other studies to explore the relative effects of biological traits of plant taxa and their distributional characteristics in the native range on invasion success [41], test broad taxonomic hypotheses about plant naturalization [42] and compare naturalization rates in the exotic floras of Australia and New Zealand (RP Duncan, RP Randall, PA Williams, unpublished data). Plant taxa in the dataset used for the SOM analysis were coded as a weed for a specific region if they were recorded in at least one of the following four descriptive categories; Weed, Noxious Weed, Agricultural Weed, Environmental Weed. Similar to Pyšek et al. [41], we have taken the categorization of a plant taxon as a ‘weed’ by the consulted sources to indicate that it was in an advanced stage of the invasion process and putatively having a negative impact [4] at the time the record was made. This is in contrast to the categories Casual Alien, Naturalized, Cultivation Escape, Garden Escape, Contaminant and Quarantine Weed also found in the database that could imply an earlier stage of invasion (e.g. casual alien or naturalization) or provide other information about the behaviour of the plant and its relationship with people.

To assess the value of the SOM approach we selected Australia as a case study and extracted results for the different Australian states and territories and generated two lists. The first list shows those plant taxa that are currently absent from Australia and ranks them by their likelihood of becoming weeds in each of the states or territories should they be introduced. The second list ranks those plant taxa that are already present somewhere in Australia by their likelihood to become weeds in the states or territories where they are not currently recorded as such. We discuss results in light of the limitations of the dataset used for the analysis and make recommendations to increase robustness of the approach.

## Methods

### Data

The data used in this study were extracted from a pre-existing, extensive plant database [39] consisting of a compilation of plant taxa cited as weeds or other related status in at least one region based on consultation of approximately 1,100 references from around the world, which provides a reasonable global coverage [41]. A total of 165,283 entries (taxon-region combinations) with at least one record in one or more of the descriptive status categories were extracted from the database.

Entries associated with no particular region or with very large regions (e.g. global, pantropics, Northern Hemisphere, South America, Africa, Asia, Europe) were deleted from the dataset. Wherever appropriate, the regions of the dataset were reformatted according to the geographical regions of the United Nations Statistics Division [43]. For the purpose of the analysis, the following modifications were also made: 1) the region ‘Pacific’ was retained even though the United Nations considers it as three regions (Melanesia, Micronesia and Polynesia), 2) entries for the Middle East were placed under Western Asia, which contains all countries that are typically comprised in this political region (except Iran), 3) entries for the Republic of Korea (South Korea) and for Korea were grouped and placed under the latter name; 4) a region administratively linked to a country that is geographically distant was grouped with the closest geographical region (e.g. Hawai'i nested within Pacific, Reunion Island nested within Eastern Africa, Canary Islands nested within Northern Africa, Svalbard nested within Northern Europe, Northern Ireland changed to Ireland (north) and Ireland changed to Ireland (south) with both nested within Ireland (south & north) and Northern Europe, Christmas Island nested within South Eastern Asia). Some countries were further subdivided into their states, provinces, or other regions (e.g. islands). The 1,971 entries associated with South-Eastern Australia in the extracted dataset were duplicated and allocated to each of the state of New South Wales (NSW; which encompasses the Australian Capital Territory) and Victoria. These were in addition to entries that were specific to each of these states (2,723 for NSW and 3,173 for Victoria). Several geographical sub-regions were created for the USA within which the various states were nested based on geography.

Entries without full name of plant taxon and details such as sub-species and cultivar names were deleted from the dataset. Hybrid names were retained only when the parent names were included in the records. Pairs of names that were very similar were checked for spelling and corrected.

For each region, an entry was coded with a ‘1’ (i.e. considered a weed) when the taxon was recorded in one or more of the four descriptive status categories that explicitly indicate that a taxon is considered a weed in that particular region (Weed, Noxious Weed, Agricultural Weed, Environmental Weed). Entries recorded in the other categories (Casual Alien, Naturalised, Cultivation Escape, Garden Escape, Contaminant, Quarantine Weed) were coded with a ‘0’ and not considered as weeds. A definition of each of these categories of the original database is provided in supplementary information (Table S1). It is noteworthy that while the category Quarantine Weed contains the word ‘weed’, entries against this category were not coded with a ‘1’ because it refers to plant taxa that are not wanted and not yet present in the region where they are recorded as such. It is important to note that ‘0’ does not necessarily indicate absence from a region. A plant taxon could be absent, but it could also be present, just not recorded in the selected ‘weed’ categories (hereafter simply referred to as ‘recorded as a weed’) in that region. Identifying plant taxa that are absent from a region requires consultation of the relevant flora or database of native and introduced plant taxa for the specific region (as we have done for Australia – see below).

Plant taxa in the reformatted dataset that were not recorded as weeds in any region (i.e. coded with ‘0’) were deleted prior to analysis. We were computationally limited by the number of taxa that could be included in the analysis and so also removed taxa that were only recorded as weeds in one or two regions. Taxa with so few weed records are unlikely to receive a high SOM index (see below) and to rank highly in any subsequent list. Upon removal of these taxa, we found that 46 of the 233 regions of the dataset did not contain any weed records. These regions were removed as they provided no data for the SOM analysis and could not therefore be reliably evaluated. The final dataset used for the analysis was a matrix comprising 187 vectors (regions) (Table S2), each with 6690 elements (plant taxa), where each element of a vector represented whether a plant taxon in a geographical region is recorded as a weed (1) or not (0).

### SOM analysis

A SOM is an artificial neural network composed of neurons in a regular lattice structure, which is able to convert high dimensional data into a two dimensional map representing the similarity between data points (in this case, geographic regions). Those data points found close together on the map are most similar to each other [44]. The number of neurons in a SOM is partially determined by the heuristic rule suggested by Vesanto et al. [45], which is 5√n, where n is the number of samples. In addition, the two largest eigenvalues are calculated from the data set and the ratio of length and width of the SOM is set to those eigenvalues. Given this ratio, the final number of neurons is set as close to Vesanto's heuristic rule as possible. The map size used in this analysis was 12×6 (72 neurons) with the standard hexagonal configuration and the recommended number of iterations: 36000 [44]. Full details describing a SOM analysis can be obtained from Worner and Gevrey [35] or Kohonen [44], but essentially, each of the 187 regions in this dataset occupies a point in space of 6690 dimensions. Each region's position in this multidimensional space is determined by the 6690 element vector that describes whether each plant taxon is recorded as a weed or not in that region. The SOM projects its 72 neurons into this space via neuron weight vectors. As with the region vectors, these neuron weight vectors are composed of 6690 elements, which describes each neuron's point in this multidimensional space. While this initial projection of neurons into the multidimensional space can be random, we use a linear initialization that distributes the neurons corresponding to the first two eigenvalues discussed above. This linear initialization distributes the neurons in a way that is more representative of the raw data and significantly reduces the time taken to train the network and complete the analysis [44].

When the analysis is initiated, each raw data point is assessed and the neuron closest to this data point in the multidimensional space is identified as the best matching unit (BMU). The neuron weight vector of this BMU is then adjusted, moving it closer to the data point. Because all neurons are connected in a large 12×6 ‘elastic net’, the process of moving one neuron exerts a gravitational force that drags other neurons in the SOM with it. Each raw data point is assessed simultaneously (batch algorithm) to complete one iteration. With subsequent iterations, the neurons spread out to eventually occupy approximately the same area that the data points occupy in the multidimensional space. When the analysis is completed, each data point or region vector has a BMU that is its closest neuron. Regions that have similar weed assemblages are located close together in the multidimensional space and will have the same BMU.

Each neuron weight vector comprises 6690 elements, with each element having a value between 0 and 1. Each of these elements corresponds to one of the 6690 plant taxa and can be interpreted as the strength of association of that plant taxon with the assemblage of weeds in that neuron and hence any weed assemblage of any region associated with that neuron (BMU). The SOM analysis therefore generates an index for all plant taxa describing the likelihood of that taxon becoming a weed in that particular region.

The analysis was performed using Matlab [46] and the SOM Toolbox (version 2.0) developed by the Laboratory of Information and Computer Science, Helsinki University of Technology (http://www.cis.hut.fi/projects/somtoolbox/). SOM indices were then extracted for all taxa for each of the different states and territories of Australia and the original database [39] was consulted to identify the plant taxa that are absent from Australia. Relationships between SOM indices and the number of regions a plant taxon is recorded as a weed or the number of taxa recorded as weeds in a region were also determined.

## Results

Of the 6690 plant taxa in the dataset used for the SOM analysis, 1599 are absent from Australia and 5091 are currently present in one or more states/territories. Within the 5091 taxa present in Australia, 745 (14.2%) are Australian native plant species, some of which naturalized outside their native range within the country, and the remaining are introduced taxa [39]. The number of taxa in the dataset that are recorded as weeds in Australia and the number of literature sources consulted in building the original database [39] vary considerably between each state and territory (Table 1). To provide an idea of outputs from the SOM analysis, the top 20 plant taxa, for each state (except Tasmania) and the Northern Territory, that are currently absent from Australia, but have the highest SOM indices (i.e. likelihood of becoming weeds if introduced) were extracted from the full lists of SOM indices (Tables 2, S3). Plant taxa in NSW and Victoria have the same SOM indices because these states were assigned to the same neuron in the analysis and therefore a single list is presented. Across the states/territories, 70–100% of the plant taxa in the top 20 have been recorded as agricultural weeds somewhere in the world, while only 25–45% of the taxa have been recorded as environmental weeds (Table S3).

**Table 1:** Number of taxa in the dataset that are recorded as weeds in each Australian state and territory and number of literature sources available and consulted at the time the original database was built [39].

State/territory	Number of taxa recorded as weeds	Number of literature sources
New South Wales^a^	2235	21
Northern Territory	533	5
Queensland	755	25
South Australia	126	5
Tasmania	3	6
Victoria	2011	23
Western Australia	914	23

a Includes the Australian Capital Territory.

**Table 2:** Plant taxa absent from Australia – top 20 plant taxa for each state (except Tasmania) and Northern Territory that are currently absent from Australia, but have the highest likelihood of becoming weeds if introduced (for full list see Table S3).

New South Wales & Victoria^a^		South Australia		Western Australia	
Taxon	SOM Index	Taxon	SOM Index	Taxon	SOM Index
*Myriophyllum spicatum*	0.47	*Striga hermonthica*	0.14	*Myriophyllum spicatum*	0.55
*Galinsoga ciliata* b	0.45	*Orobanche aegyptiaca*	0.12	*Striga gesnerioides*	0.53
*Potamogeton pusillus*	0.44	*Guizotia scabra*	0.09	*Gisekia pharnacioides*	0.53
*Euphorbia serrata*	0.44	*Oxygonum sinuatum*	0.09	*Hackelochloa granularis*	0.50
*Plantago virginica*	0.44	*Capsella rubella*	0.09	*Celosia trigyna*	0.49
*Stipa trichotoma*	0.42	*Trachynia distachya*	0.09	*Setaria pallide-fusca*	0.46
*Striga gesnerioides*	0.40	*Cyperus longus*	0.09	*Galinsoga ciliata* b	0.44
*Gisekia pharnacioides*	0.40	*Setaria adhaerens*	0.08	*Plantago virginica*	0.43
*Cardamine impatiens*	0.40	*Anacyclus clavatus*	0.08	*Potamogeton pusillus*	0.43
*Lappula echinata*	0.40	*Eclipta alba*	0.07	*Euphorbia serrata*	0.42
*Hackelochloa granularis*	0.40	*Striga asiatica*	0.07	*Stachytarpheta indica*	0.42
*Celosia trigyna*	0.40	*Matricaria inodora*	0.07	*Striga asiatica*	0.42
*Setaria pallide-fusca*	0.40	*Solanum dubium*	0.06	*Solanum nodiflorum*	0.42
*Myriophyllum brasiliense*	0. 40	*Diplotaxis catholica*	0.06	*Vossia cuspidata*	0.42
*Striga asiatica*	0. 40	*Diplotaxis erucoides*	0.06	*Myriophyllum brasiliense*	0.42
*Stachytarpheta indica*	0. 40	*Digitaria insularis*	0.05	*Stipa trichotoma*	0.42
*Vossia cuspidata*	0. 40	*Scirpus acutus*	0.05	*Cardamine impatiens*	0.41
*Boerhavia repens*	0. 40	*Ridolfia segetum*	0.05	*Lappula echinata*	0.41
*Hydrocotyle americana*	0. 40	*Polypogon fugax*	0.05	*Suaeda fruticosa*	0.41
*Suaeda fruticosa*	0. 40	*Ludwigia prostrata*	0.05	*Boerhavia repens*	0.41

Northern Territory		Queensland	
Taxon	SOM Index	Taxon	SOM Index
*Triumfetta semitriloba*	0.59	*Triumfetta semitriloba*	0.62
*Galinsoga quadriradiata* b	0.47	*Paspalum fimbriatum*	0.53
*Paspalum fimbriatum*	0.42	*Myriophyllum spicatum*	0.51
*Erechtites hieraciifolius*	0.42	*Gomphrena serrata*	0.51
*Caperonia palustris*	0.40	*Acalypha alopecuroides*	0.46
*Digitaria insularis*	0.40	*Blechum pyramidatum*	0.44
*Gomphrena serrata*	0.40	*Galinsoga quadriradiata* b	0.44
*Borreria alata*	0.39	*Hydrolea spinosa*	0.42
*Myriophyllum spicatum*	0.38	*Anredera vesicaria*	0.42
*Jacquemontia tamnifolia*	0.37	*Neyraudia reynaudiana*	0.42
*Heliotropium procumbens*	0.37	*Abutilon hirtum*	0.40
*Spigelia anthelmia*	0.37	*Agdestis clematidea*	0.40
*Acalypha alopecuroides*	0.35	*Launaea intybacea*	0.40
*Ipomoea asarifolia*	0.35	*Pseuderanthemum fasciculatum*	0.40
*Anredera vesicaria*	0.35	*Zeuxine strateumatica*	0.40
*Chamaecrista nictitans*	0.35	*Oxalis barrelieri*	0.38
*Panicum purpurascens*	0.34	*Caperonia palustris*	0.38
*Croton lobatus*	0.33	*Jacquemontia tamnifolia*	0.37
*Cordia curassavica*	0.33	*Heliotropium procumbens*	0.37
*Striga gesnerioides*	0.31	*Ipomoea asarifolia*	0.37

a Includes the Australian Capital Territory.

b *Galinsoga quadriradiata* is considered a subspecies of *G. caliata* by some authors.

The top 20 plant taxa for each state (except Tasmania) and the Northern Territory that are present in Australia, but not currently recorded as weeds in the particular state/territory and have the highest SOM indices (i.e. likelihood of becoming a weed in the future) were also extracted from the full lists of SOM indices (Tables 3, S4). Separate lists were generated for plant taxa that are not currently recorded as weeds in NSW and Victoria since, as expected, these taxa differ for each state. Across the states/territories, 90–100% and 75–100% of the top 20 taxa have been recorded somewhere in the world as agricultural and environmental weeds, respectively (Table S4).

**Table 3:** Plant taxa present in Australia – top 20 plant taxa for each state (except Tasmania) and Northern Territory, which are present in Australia but not currently recorded as weeds in the particular state/territory and have the highest likelihood of becoming one in the future (for full list see Table S4).

New South Wales^a^		Victoria		South Australia	
Taxon	SOM Index	Taxon	SOM Index	Taxon	SOM Index
*Bromus japonicus*	0.83	*Pennisetum purpureum*	0.79	*Cynodon dactylon*	0.56
*Rumex acetosella*	0.80	*Kochia scoparia*	0.78	*Echinochloa crus-galli*	0.47
*Ciclospermum leptophyllum*	0.77	*Phytolacca americana*	0.78	*Portulaca oleracea*	0.46
*Rubus phoenicolasius*	0.75	*Cnicus benedictus*	0.77	*Solanum nigrum*	0.44
*Rubus discolor*	0.74	*Anoda cristata*	0.77	*Chenopodium album*	0.41
*Cycloloma atriplicifolium*	0.70	*Sida spinosa*	0.77	*Datura stramonium*	0.40
*Gnaphalium purpureum*	0.70	*Malvastrum coromandelianum*	0.76	*Polygonum aviculare*	0.35
*Picris hieracioides*	0.68	*Mimosa pudica*	0.76	*Sonchus oleraceus*	0.34
*Sparganium erectum*	0.68	*Tridax procumbens*	0.76	*Anagallis arvensis*	0.34
*Hieracium praealtum*	0.68	*Datura metel*	0.76	*Eleusine indica*	0.33
*Rubus armeniacus*	0.68	*Acanthospermum hispidum*	0.76	*Bidens pilosa*	0.32
*Platanus hybridus*	0.67	*Passiflora foetida*	0.76	*Cyperus esculentus*	0.32
*Acacia melanoxylon*	0.66	*Euphorbia heterophylla*	0.76	*Sorghum halepense*	0.32
*Thymelaea passerina*	0.65	*Bidens bipinnata*	0.76	*Poa annua*	0.30
*Rhynchelytrum repens*	0.63	*Mangifera indica*	0.76	*Galium aparine*	0.28
*Polygonum capitatum*	0.63	*Pereskia aculeata*	0.76	*Echinochloa colona*	0.27
*Cryptostegia grandiflora*	0.62	*Zea mays*	0.75	*Capsella bursa-pastoris*	0.25
*Eucalyptus saligna*	0.62	*Psidium guajava*	0.75	*Digitaria sanguinalis*	0.25
*Eclipta prostrata*	0.61	*Onobrychis viciifolia*	0.75	*Stellaria media*	0.24
*Agropyron repens*	0.61	*Saccharum officinarum*	0.75	*Setaria italica*	0.23

Western Australia		Northern Territory		Queensland	
Taxon	SOM Index	Taxon	SOM Index	Taxon	SOM Index
*Oxalis corniculata*	0.83	*Thunbergia alata*	0.82	*Leonotis nepetifolia*	0.89
*Brassica juncea*	0.81	*Cardiospermum halicacabum*	0.79	*Mimosa pigra*	0.81
*Thunbergia alata*	0.78	*Myriophyllum aquaticum*	0.78	*Rottboellia cochinchinensis*	0.78
*Taraxacum officinale*	0.78	*Paspalum urvillei*	0.78	*Cryptostegia madagascariensis*	0.73
*Amaranthus spinosus*	0.77	*Plantago major*	0.78	*Commelina diffusa*	0.72
*Crotalaria pallida*	0.76	*Momordica charantia*	0.77	*Youngia japonica*	0.72
*Mirabilis jalapa*	0.76	*Achyranthes aspera*	0.76	*Rhynchelytrum repens*	0.70
*Eragrostis pilosa*	0.76	*Egeria densa*	0.74	*Sporobolus indicus*	0.69
*Macfadyena unguis-cati*	0.74	*Physalis angulata*	0.73	*Hydrilla verticillata*	0.69
*Lolium perenne*	0.74	*Hedychium coronarium*	0.73	*Melia azedarach*	0.69
*Helianthus annuus*	0.74	*Urena lobata*	0.73	*Psidium cattleianum*	0.69
*Passiflora edulis*	0.74	*Chenopodium ambrosioides*	0.69	*Imperata cylindrica*	0.68
*Psidium guajava*	0.73	*Commelina diffusa*	0.68	*Mirabilis jalapa*	0.67
*Eriobotrya japonica*	0.73	*Impatiens walleriana*	0.68	*Taraxacum officinale*	0.67
*Jacaranda mimosifolia*	0.73	*Lonicera japonica*	0.67	*Alternanthera sessilis*	0.67
*Ligustrum sinense*	0.73	*Mirabilis jalapa*	0.67	*Sphenoclea zeylanica*	0.67
*Setaria italica*	0.73	*Rhynchelytrum repens*	0.67	*Clerodendrum philippinum*	0.67
*Melia azedarach*	0.73	*Bidens alba*	0.65	*Triphasia trifolia*	0.66
*Rottboellia cochinchinensis*	0.72	*Asystasia gangetica*	0.65	*Sonchus asper*	0.65
*Daucus carota*	0.72	*Eclipta prostrata*	0.64	*Lindernia crustacea*	0.65

a Includes the Australian Capital Territory.

For both lists, it is noteworthy that the SOM indices for South Australia are considerably lower than those for any other Australian state or territory. SOM indices for Tasmania are not presented because the final dataset for that state contained only three plant taxa recorded in one or more of the four categories explicitly including the word ‘weed’ and little confidence could be shown in this state's list [38].

**Figure 1:**
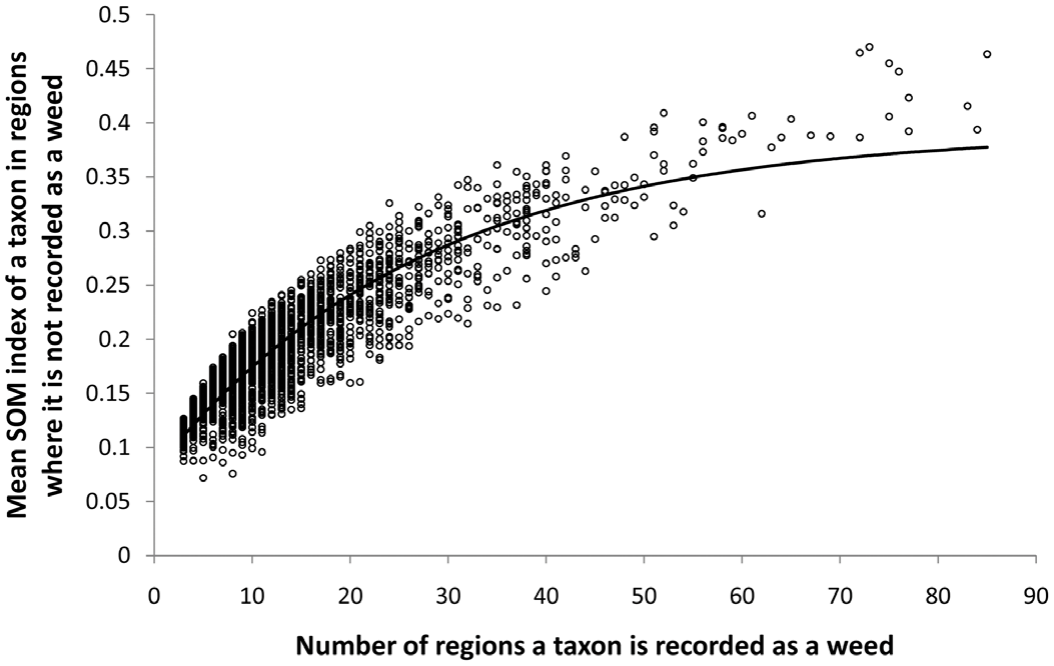
A weed taxon's distribution and its mean SOM index. Relationship between the number of regions a plant taxon is recorded as a weed and the mean of SOM indices (i.e. likelihood of becoming a weed) given to that taxon in all regions where it is not recorded as a weed (arc sine transformed).

**Figure 2:**
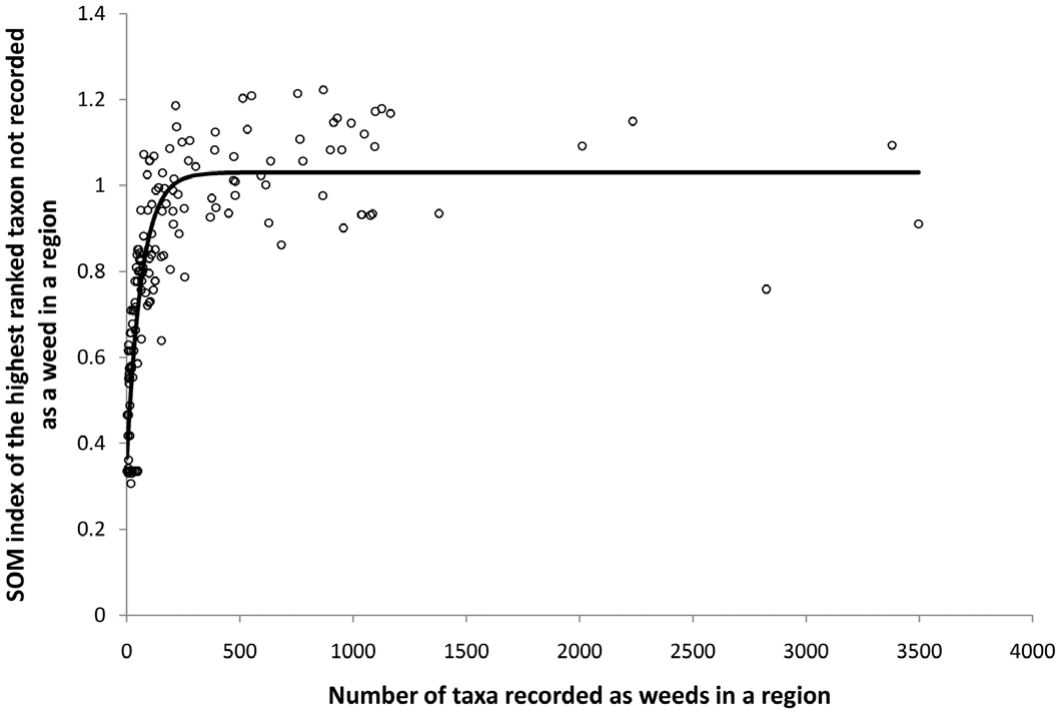
A region's weed taxa and SOM index of the highest ranked taxon. Relationship between the number of taxa recorded as weed in a region (arc sine transformed) and the SOM index (i.e. likelihood of becoming a weed) for the highest ranked taxon that is currently not recorded as a weed in that region.

A significant positive, but decelerating relationship was observed between the number of regions a plant taxon is recorded as a weed and the mean of SOM indices given to that taxon in regions where it is not recorded as a weed (non-linear regression, F_2,6688_ = 21442.19, p<0.001, R^2^ = 0.865, response data arc sine transformed) (Fig. 1). A positive relationship was also observed, up to a threshold of approximately 300 taxa, between the number of plant taxa recorded as weeds in a region and the SOM index of the highest ranked taxon that is not recorded as a weed in the same region (non-linear regression, F_2,184_ = 447.47, p<0.001, R^2^ = 0.839, response data arc sine transformed) (Fig. 2)

## Discussion

A SOM analysis is an attractive analytical technique because it can perform a robust simultaneous analysis of a large number of species, is free of human biases, and resilient to significant errors in the dataset [37], [38]. Its use in ecology has so far been restricted to predicting establishment of species in new areas based on analysis of presence and absence data in regions across the globe [34], [35], [36], [37], [38]. In this study, we have applied the SOM approach to a dataset extracted from a pre-existing global plant database [39] to predict weediness of plant taxa before and after they have been moved to a new region. Assessing the potential of this analytical approach and identifying pitfalls relating to the dataset used was facilitated by focusing on results obtained for Australia, our case study.

It is important to emphasize that results from a SOM analysis of plant taxa would not be intended for use in isolation, but rather for initial screening to provide complimentary information during expert solicitation processes to identify plant taxa that have the highest likelihood of becoming weeds in the future [37]. Results could also guide subsequent decisions as to which taxon should be investigated further using approaches such as weed risk assessment, niche modelling and cost-benefit analysis. The SOM indices for plant taxa that are currently absent from a region could be used to guide discussions on which taxa should be listed for national surveillance to achieve successful early detection, such as the Category 1 taxa comprised in the recently developed national categorization system for invasive species by the Australian government [47]. On the other hand, SOM indices for taxa that are already present in a region, but not currently recorded as weeds, could contribute to decision making regarding the prioritization of early management interventions to prevent future problems. More importantly, the SOM indices could provide a first screen of plant taxa prior to undergoing more systematic analysis through, for example, a post-border weed risk management protocol [48].

Overall, the SOM analysis revealed that plant taxa already recorded as weeds in a large number of regions tend to have higher SOM indices, or likelihood of becoming weeds, in regions in which they are not currently recorded as weeds. This observation was not surprising since a SOM analysis is the quantitative equivalent of asking whether a plant taxon is a weed somewhere else, which has already been identified as the most valuable attribute for predicting weediness in a new region [16], [22], [49]. A SOM however, goes further by also assessing plant taxa associations. In other words, the SOM evaluates if a taxon is a weed somewhere else, what other taxa are weeds at that location and if those taxa are also recorded as weeds in the region of interest.

In both lists of plant taxa generated for the Australian states and territories, the SOM indices for South Australia are overall much lower than those for the other states/territories. This is a reflection of the fewer number of taxa recorded in one or more of the four selected categories containing the word ‘weed’ in this state. There is a positive relationship between the number of taxa recorded as weeds in a region (up to approx. 300) and the SOM index of the highest ranked taxon that is not recorded as a weed in the same region. South Australia has only 126 taxa recorded as weeds in the dataset, while considerably higher numbers are recorded in the other states and the Northern Territory. In other words, the SOM assesses a taxon's ‘strength’ of association with the weed taxa assemblage present in a region and consequently the more taxa recorded as weeds in that region the stronger that association is likely to be.

SOM indices generated for Tasmania are not presented because a region with such a small number of taxa recorded in one or more of the four selected categories containing the word ‘weed’ in this dataset, in this case only three taxa, significantly reduces the confidence in taxa rankings by SOM [38]. The low number of plant taxa recorded as weeds in the dataset for Tasmania as well as for South Australia, albeit to a lesser extent, was surprising. This may be explained by the lower number of relevant literature sources for these two states available at the time the original database was built [39] compared to that available for the other states/territories. It is noteworthy that while only five sources were available for the Northern Territory, two of these were considerably more comprehensive than any of the five and six sources available for South Australia and Tasmania, respectively. Furthermore, discrepancies in the way different literature sources have categorized plant taxa in different states/territories cannot be ruled out. For example, over 99% of the Tasmanian introduced flora in the database is classified in categories that do not include the term ‘weed’ [39].

It is noteworthy that the state/territory lists of SOM indices for the top 20 plant taxa that are present in Australia, but not currently recorded as weeds in the particular state/territory contain some startling rankings. For example, the listing of the tropical and sub-tropical species *Mangifera indica* (mango) and *Saccharum officinarum* (sugarcane) in the top 20 taxa with the highest likelihood of becoming weeds in Victoria is a surprising result. NSW and Victoria have very similar assemblages of plant taxa recorded as weeds (84.6% similarity) and were therefore assigned to the same neuron in the analysis, thus generating the same SOM indices for both states. This grouping however, does not take into consideration the range of climatic conditions that significantly differ between these two states. Both species are recorded as weeds in NSW, which has a subtropical climate in its northern coastal region, but not in Victoria where there is not likely to be a suitable climate for them to thrive. Nonetheless they were assigned a high SOM index during the analysis, which explains why they are both listed in the top 20 taxa with the highest likelihood of becoming weeds in Victoria. A SOM analysis of weed assemblages at a finer scale would reduce the chance of obtaining unusual rankings such as this (though some care would need to be taken to avoid a significant increase in false negatives). However, state level data is the best available scale at present.

For each state/territory, the SOM indices or likelihood of becoming a weed for the taxa absent from Australia are overall much lower than those for taxa that are already present in Australia. These results were not surprising considering the positive relationship that has been identified between SOM indices and the number of regions a taxon is recorded as a weed. The mean number of regions in which the 20 taxa absent from Australia with the highest SOM indices have been recorded as weeds is 10.26, while it is 33.45 for the top 20 taxa present in Australia. Further, many of the taxa with high SOM indices that are present in Australia but not currently recorded as weeds in a particular state/territory are actually recorded as a weed in one or more other state/territory that are either clustered within the same neuron or a neighbouring neuron in the SOM two dimensional map. This observation further supports the conclusions of Paini et al. [36] and Randall [50] that the greatest threat comes from taxa already present within a country.

One of the difficulties in applying the SOM approach to plant taxa has been the multitude of orthographic variants for names, including feminine and masculine forms, as well as spelling errors that are widespread in the literature sources consulted to build the database mined for this study [39]. Wherever possible, names were standardized and corrected in the extracted dataset prior to undertaking the analysis, but it is likely that some errors remain considering the thousands of plant taxa in the dataset. Nevertheless, these errors may not matter since the SOM method has been shown to adequately handle an error rate of up to 20% in the dataset before rankings are considerably altered [37]. It was not possible however, within a reasonable timeframe to systematically review all 6,690 names of the dataset to make sure that the current taxonomic name with broad consensus from the scientific community was used for each taxon and that all records of synonyms were agglomerated. For example, *Xanthium strumarium* is a variable species comprising several subspecies and varieties, which is widely referred to in floras in the Americas and Europe [51]. Some, but not all, taxonomists however, claim that the *X. strumarium* complex can be divided into a number of different species, including *X. occidentale* which is commonly referred to as Noogoora burr in Australia [52]. The dataset analyzed comprised both *X. strumarium* and *X. occidentale*, which are reported to be present in Australia, and each was given different SOM indices in each of the states/territories. Interestingly, Queensland is the only state/territory in which *X. strumarium* has not been recorded as a weed (ranked 27^th^ most likely to become a weed), which indicates the name *X. occidentale* is solely used in that state. This example highlights the importance of carefully interpreting results from the SOM analysis presented in this paper.

The key and most important limitation in using a SOM analysis to predict weediness of plant taxa intrinsically lies with the dataset used. The inclusion of a plant taxon in one of the four categories extracted from the pre-existing database mined for our study, which explicitly indicates that a taxon is considered a weed in a particular region (Weed, Noxious Weed, Agricultural Weed, Environmental Weed), simply reflects that someone has reported the taxon as such in the sources consulted to build the database. The classification of a taxon as a weed in the literature is not often based on actual data that document the economic, environmental and/or social impact of the taxon, but mostly based on human perceptions that the taxon is troublesome or simply that it is not wanted in a particular situation [4], [5]. For example, of the 5789 terrestrial plants alien to Europe included in the DAISIE database, ecological and economic impact has been documented for only 5.6 and 5.4% of the taxa, respectively [53]. Determining the impact or consequences of a plant taxon at a site where it is not wanted can be problematic because most species are often only noticed after a prolonged period of time and it is based on variable human economic, social, aesthetic and environmental values [28].

While a SOM analysis of global weed assemblages would inject a much needed level of objectivity in assessing the risk of plant taxa becoming weeds, it will have to wait until a worldwide dataset of plant taxa based on agreed terminology and a uniform categorization scheme is developed [5], [14], [54]. The adoption of consistent and objective criteria that incorporate a standardized approach for impact assessment of plant taxa would considerably improve our ability to develop an appropriate dataset from which to draw generalizations and make more robust predictions regarding weediness. The internet and social media, which are increasingly being used around the world, could become the conduit to enlist interested parties in gathering the necessary information to build such extensive global dataset, pending an agreement on definitions can be reached and applied uniformly. To address the complex issue of synonyms, the web-based template for entering data could be linked to one or more recognized databases of plant names (e.g. Germplasm Resources Information Network – (GRIN); International Plant Names Index (IPNI)) to ensure that all names under which a taxon has been known are automatically listed for each entry.

It would probably be more realistic in the short to medium term to concentrate efforts into developing a global dataset of plant taxa that fit the characteristics of invasive in specific regions, as defined by Richardson et al. [4]; i.e. reproduce consistently and prolifically, without or in spite of human intervention, and spread at considerable distances from parent plants, without any inference to environmental and economic impact. Determining if a taxon possesses these characteristics would be less prone to bias from human perceptions and value systems, as is typically the case when deciding, without some level of research, if a plant is harmful and having a negative economic, environmental or social impact. Performing a SOM analysis on such a dataset would generate predictions as to the likelihood of plant taxa becoming invasive in new regions. Such predictions would be useful for planning by policy makers and land managers considering the substantial time lag that exists between introduction, naturalization and invasiveness of plant taxa [55].

Predicting the invasiveness and future impact of a taxon introduced in a new region will remain a challenge because it not only depends on the taxon but also on the ecological properties, natural disturbances and management practices of the recipient land [56]. Nonetheless, the application of predictive tools, such as SOM and Weed Risk Assessment, may be the key for policy makers and land managers to make informed decisions regarding weeds and act before problems get out of hand.

## Supporting Information

Table S1 **Definition of each descriptive status category of records extracted from the original, pre-existing database to build the dataset for the SOM analysis.**
(XLS)

Table S2 **Regions comprised in the final dataset analysed.**
(XLS)

Table S3 **SOM indices of plant taxa in each state (except Tasmania) and the Northern Territory that are currently absent from Australia, but have the highest likelihood of becoming weeds if introduced.**
(XLSX)

Table S4 **SOM indices of plant taxa in each state (except Tasmania) and the Northern Territory that are present in Australia, but not currently recorded as weeds in the particular state/territory and have the highest likelihood of becoming one in the future.**
(XLSX)
